# The effect of indoor office environment on the work performance, health and well-being of office workers

**DOI:** 10.1186/s40201-014-0113-7

**Published:** 2014-08-09

**Authors:** Komalanathan Vimalanathan, Thangavelu Ramesh Babu

**Affiliations:** Department of Mechanical Engineering, Kingston Engineering College, Vellore, India; Department of Industrial Engineering, Anna University, Chennai, India

**Keywords:** Office workers, Office environment, Health, Well-being, Performance

## Abstract

**Background:**

The effect of indoor environment may have an influence on the performance, productivity health and well-being of office workers.

**Methods:**

Environmental factors such as indoor temperature and illumination have been investigated at three levels. A neurobehavioral test (NBT) has been proposed for the evaluation of office workersc performance. A field lab to emulate an office has been created. In controlled condition of environmental factors, the neurobehavioral test was conducted. The response time and the number of errors in each test have been recorded. A randomized block factorial design was used to analyze the responses of office worker’s performance.

**Results:**

The results revealed that the independent and interaction effect of temperature and illumination have significant effect on the office workers’ performance. The effect of indoor room temperature has more influences than the effect of illumination. The effect of indoor temperature has 38.56% of contribution on the performance. The optimum levels of indoor temperature at 21°C and illumination at 1000 lux have improved the work performance and health of office workers.

**Conclusion:**

The indoor room temperature and illumination are more influence on the performance of the office workers. It may be concluded that the impact of indoor room temperature (38.56%) is more on the office worker’s performance than the effect of illumination (19.91%). Further, it may be concluded that the optimum level of indoor room temperature (21°C) and illumination (1000lux) have improved the work performance, health and productivity of office workers.

## Introduction

An office is a place where professional duties and administrative work are carried out in the organization building. The work depends on the type of business, but it will usually include using computers, communicating with others by e-mail, telephone or fax, keeping records and files etc., in hard and soft format. Features of an office such as people, building space, equipment, furniture and the environment, must fit together well for workers to feel healthy and comfortable and to be able to work efficiently**.** More than 50% of the world’s population currently works in some form of office. Mostly the developing countries likes India and China are having more population. They are working with machines and majority of them are from computer related sector. In the Information Technology (IT) and Information Technology Enable Services (ITES), workers are dependent on the computers. More IT and ITES sectors are increasing in India. The study on performance, health and well-being of office workers productivity is an essential to improve it. Indoor environmental quality has an important role to play in office workers’ performance, health and well-being. The effect of environmental factors brings down the health and efficiency of office workers. The primary objectives of this study are to improve human health, safety, and improve performance. Any study on these environmental factors can potentially benefit millions of people around the world. Hence an attempt has been made to carry out a study on the performance of office workers by considering the indoor office environment.

An essential requirement of office workers’ performance and productivity improvement is indoor environmental quality. The indoor room temperature and illumination are the most important vital factors that affect the performance of office workers. The thermal discomfort caused by elevated air temperature had affected the performance of office workers. Indoor environmental quality has influence on the office workers Performance [1]. The office workers spend 90% of the time in indoor environment. Indoor room environment has direct relation with the office worker’s health and well-being. 10% of office worker’s Performance may be increased by achieving the best indoor environmental quality [2]. The office workers had more negative emotions and had to use more effort to maintain performance under slightly warm or slightly cool environment conditions [3]. Environmental factors have imperative role to play in the effectiveness of office workers. A neurobehavioral approach [4] had been proposed to evaluate the effect of office indoor room temperature and illumination on the office worker’s Performance. The NBT involves all the neurobehavioral functions such as emotion, perception, learning and memory, thinking and execution function. The performance of NBT decreased when the thermal condition in the indoor room was deviated from the neutral conditions. While comparing with neutral condition, the performance decreased at the slightly cool or slightly warm environment condition [1]. The productivity is one of the most important factor which can affect overall performance of any organization either small or entire nation. The Performance of call center workers has less Performance, when the temperature was above 25°C. Performance has been reduced to 2.4% per degree temperature increase between 21.9°C to 28.5°C. Similarly call center worker’s Performance was reduced 5 – 7% while the indoor room temperature exceeds 25°C [5]. Federspiel et al. [6] has measured the Performance of call center workers in the US. Within the comfort zone, varying the room temperature had no significant effect on Performance of the workers. Indoor room temperature at more than 25.4°C was affected the performance of office worker. During the high indoor room temperature, the lower work performance was shown by the office worker.

Charles et al. [7] has stated that indoor air quality and thermal comfort are most important factors for the worker’s performance, satisfaction and well-being. Poor office environmental conditions can affect the worker’s visual discomfort and thermal discomfort. This may affect the health and well-being of workers. Very low and very high indoor room temperature and humidity can dissatisfy the workers and also create health problems. Air conditioned office aim to provide a thermally accepted environment for office worker’s comfort and health [8,9]. Henri et al. [10] clearly explain that proper lighting was an important factor that influence on the Performance. The effect of increased or decreased illumination affects the performance, psychological and biological effect of workers. The employees preferred high illumination rather than low. Pilcher et al. [11] has reported that very hot and cold temperature conditions had effect on the performance of workers. These room temperatures have negative impact on a wide range of cognitive related task. In cold condition [10°C] the workers performance had decreased at an average of 13.91%. Similarly in hot condition [32.22°C], the performance had reduced at an average of 14.88%.

Hiroshi et.al. [12] has reported that the higher illumination has significant effect on the task performance of office workers Performance. More than 9% performance improvement was achieved in higher illumination. Parsons [13] has stated that the study of workers response on environmental factors has an important role to play in office Performance. The thermal response of the body has consequences for the workers health, comfort and efficiency. As heat stress increases there would be an effect on mental performance. Similarly the effect of cold on human performance also have significant effect. Young S. Lee et al. [14] has explained that the indoor air quality enhance worker’s job performance in enclosed private offices. Good indoor air quality and light have more significance on the performance of office workers.

**Figure 1:**
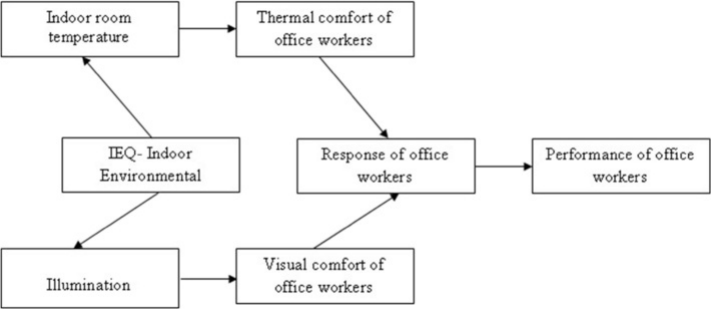
**Relation between indoor environment quality on performance.**

Environmental factors have more related influence on performance of office workers. Good working environment is an essential requirement for the office workers. Indoor temperature, illumination are the factors which is affecting the indoor environmental quality (IEQ) of an office. If IEQ affected in the office, the response of office workers will also be affected. This leads to the negative performance of office worker. So the Performance of office workers also decreased. The relation between indoor environmental factors on Performance of office worker is shown in the Figure 1.

Earlier researchers had given enough contribution to physical factor and work place design that affecting performance of office workers. The study on indoor room temperature on the performance of workers has been studied by researchers. Similarly the effect of illumination also studied already. The combined effect of environmental factors such as temperature and illumination on the performance of office workers has to be studied. Hence indoor room temperature, illumination have been investigated in this paper.

### Methodology

**Figure 2:**
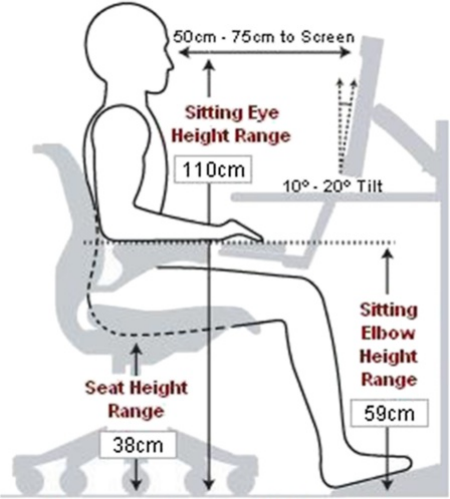
**Ergonomically designed VDT work station.**

A field lab to emulate an office has been created. The experiment was carried out in the field lab (L × W × H = 13 × 9 × 5 m) which has a controlled condition of indoor office environment. Ten voluntary participants (10 men) sat in the ergonomically designed VDT workstations (Figure 2). Each work station had a table, a chair, and a personal computer of equal configuration (Intel Pentium(R) dual CPU, E2200 @ 2.20 GHz processor 1 GB RAM). USB optical mouse and multimedia keyboard was connected with USB2.0 of the personal computer. The workstation specifications are set based on height of the individual volunteer.

For example a person with 157 cm height requires the following specifications:

The mean distance between eyes to screen was 60 cm

Seat (revolving and height adjustable) height from the floor was 38 cm

Sitting eye height from floor was 110 cm. Sitting elbow height was 59 cm

Angle of LCD monitor was 10° - 20°^.^

Necessary software had been installed before the commencement of the training. The field lab room temperature has been controlled by air conditioner. Temperature may be adjusted from 17°C to 28°C using the controls available in the air conditioner. Illumination has been controlled by regulator for focus mercury lamps and adjusting number of fluorescent lamp using on/off switch. By this way illumination may be adjusted from 500 lux to 1000 lux. The illumination level was tested at the work station’s keyboard.

### Participants

Ten undergraduate engineering students were trained in the field lab and used as office workers. Participants are all of same age group (18 years old). Wages have been given to all participants at a fixed rate per hour. To increase the performance of the participants a financial appreciation was given depends on their performance. Among the participants, whoever completed the task in minimum duration with minimum error was given an incentive at the rate of double the wages. All the participants were advised to take rest during previous night. Before the commencement of the experiments the participants have given their consent for their participation. All procedures were approved by the institutional ethics committee of Sri Balaji Chockalingam Engineering College, Arni, Tiruvannamalai District, Tamilnadu, India.

All participants in the field lab sat as per the schedule shown in Table 1. First two hours one temperature level (A_1_) was fixed and illumination levels (B_1_/B_2_/B_3_) has been changed in each 40 minutes. When beginning of each treatment combination, 10 minutes adaptation time (ADT) has been given to the participants. After the adaptation time the neurobehavioral test was conducted as per the schedule. One hour lunch break was given to all the participants.

**Table 1:** **Time duration of an experiment day**

	**ADT**	**A**_**1**_ **B**_**1**_	**ADT**	**A**_**1**_ **B**_**2**_	**ADT**	**A**_**1**_ **B**_**3**_	**ADT**	**A**_**2**_ **B**_**1**_	**ADT**	**A**_**2**_ **B**_**2**_	**ADT**	**A**_**2**_ **B**_**3**_	**Break**	**A**_**3**_ **B**_**1**_	**ADT**	**A**_**3**_ **B**_**2**_	**ADT**	**A**_**3**_ **B**_**3**_
		**Test**		**Test**		**Test**		**Test**		**Test**		**Test**	**Lunch**	**Test**		**Test**		**Test**
9 AM	9.10	9.40	9.50	10.20	10.30	11.00	11.10	11.40	11.50	12.20	12.30	1.00	2.00	2.30	2.40	3.20	3.30	4 PM

Time of the day (12 hours clock).

Legend: ADT- Adaptation time – 10 min.

Test – Neurobehavioral Test – 30 min.

A_1_B_1,_ A_1_B_12,_ A_1_B_1,_ A_1_B_2,_ etc., - Treatment level of combinations.

### Neurobehavioral test

In our research work, Neurobehavioral approach was adopted to evaluate the effect of indoor temperature, illumination on the office worker’s performance. We have computerized the neurobehavioral test [15]. This test consists of twelve parts. They were letter search, direction, object overlapping, memory span, picture detection, figure-digit, logical sequences, comprehensive reading, numerical addition, logical conclusion, picture match and reasoning. These tasks are implemented in .NET computer language.

Letter search was perception based visual search. Direction was perception based visual and hand reaction test. Object overlapping was perceptional spatial orientation test. Memory span was a concentration and memory recall test. Picture detection was a learning and memory test. Figure -digit was modalities test for checking the learning and memory. Logical sequences were thinking and executive function test. Comprehensive reading was thinking and executive function test. Numerical addition was mathematic procedures, response test. Logical Conclusion was conditional conclusion test. Picture match was thinking and executive function test**.** Reasoning was logical test. The above mentioned twelve tests were conducted.

### Measurement

Temperature and relative humidity has been measured by digital hygrometer. Illumination was measured by digital lux meter. The performance measurement was taken by recording reaction time and errors made in the test. The reaction time or time taken to complete the each test can be retrieved from the database folder of respective neurobehavioral test. Similarly the number of errors made during the test also taken from the respective database folder. The illumination was measured at the keyboard in the VDT workstation of each participant. Indoor temperature and relative humidity has been randomly measured near to each work station.

### Experiment procedure

Indoor temperature, relative humidity in% and illumination has been measured every 15 minutes in the field lab. Two measurement data have been recorded in one test duration (30 minutes). Relative humidity was recorded for the understanding relationship between indoor room temperature and relative humidity. The results of this will not be discussed in this paper. The Time taken to complete the tasks has been measured by the timer set in the computer programme. All the 10 participants were present on the day. The different combination of indoor room temperature (17°C, 21°C, 28°C) and illumination levels (500, 750, 1000 lux) has been set in the field lab. The illumination and temperature were recorded during the test conducted for each treatment combinations. Treatment combinations were randomly selected. The response time (Y_1_), number of errors (Y_2_) has been recorded in the Tables 2 and 3 respectively.

**Table 2:** **Reaction time obtained in seconds (output: response time – Y**_**1**_ **)**

**Subject**	**A1**			**A2**			**A3**		
	**B1**	**B2**	**B3**	**B1**	**B2**	**B3**	**B1**	**B2**	**B3**
S1	754	872	755	716	764	644	884	799	752
S2	784	901	705	702	732	630	850	728	766
S3	880	919	807	742	843	734	900	728	786
S4	828	897	795	808	761	658	871	701	768
S5	841	808	758	709	728	605	754	774	705
S6	823	890	782	706	746	546	766	775	769
S7	903	922	814	740	799	749	919	837	790
S8	850	880	766	702	746	693	859	780	757
S9	858	890	775	713	750	707	880	794	760
S10	902	930	815	760	794	758	915	845	803

**Table 3:** **Number of errors obtained (output: number of errors – Y**_**2**_ **)**

**Subject**	**A1**			**A2**			**A3**		
	**B1**	**B2**	**B3**	**B1**	**B2**	**B3**	**B1**	**B2**	**B3**
S1	18	19	15	19	19	15	15	17	22
S2	17	18	21	20	22	17	19	22	27
S3	19	21	20	21	22	19	23	24	29
S4	18	22	23	23	21	17	20	27	30
S5	21	25	22	22	23	17	23	25	28
S6	23	27	29	21	23	16	21	27	29
S7	24	22	24	20	22	20	23	29	33
S8	25	24	25	20	23	17	21	27	35
S9	24	30	28	21	21	19	20	28	37
S10	27	31	22	19	21	15	23	30	35

### Data collection and analysis

**Figure 3:**
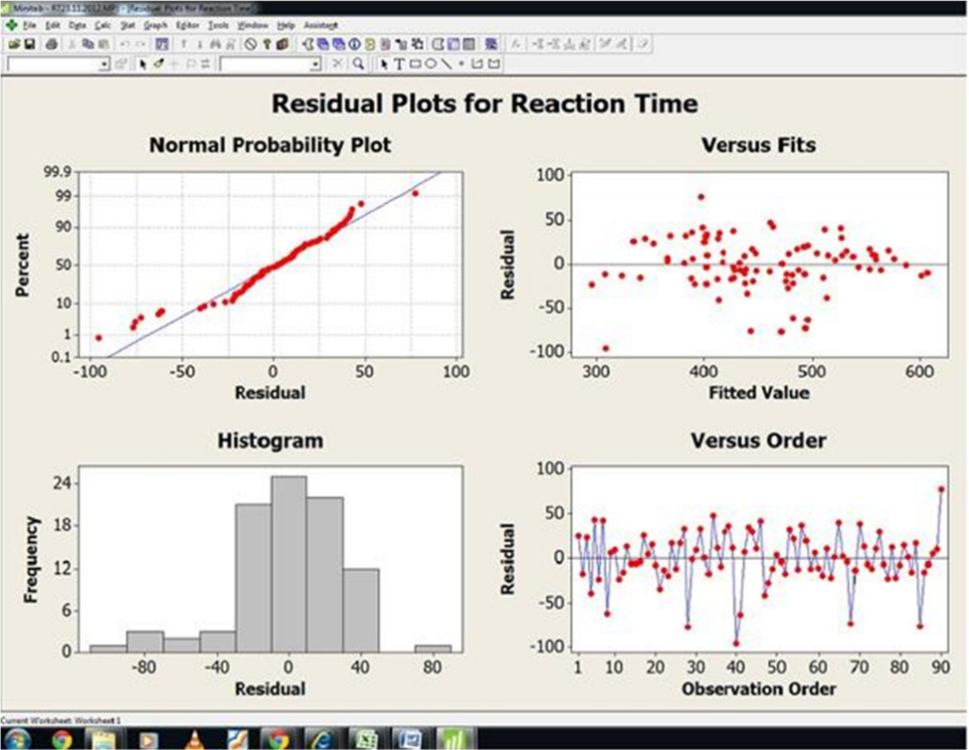
**Residual plot for reaction time.**

**Figure 4:**
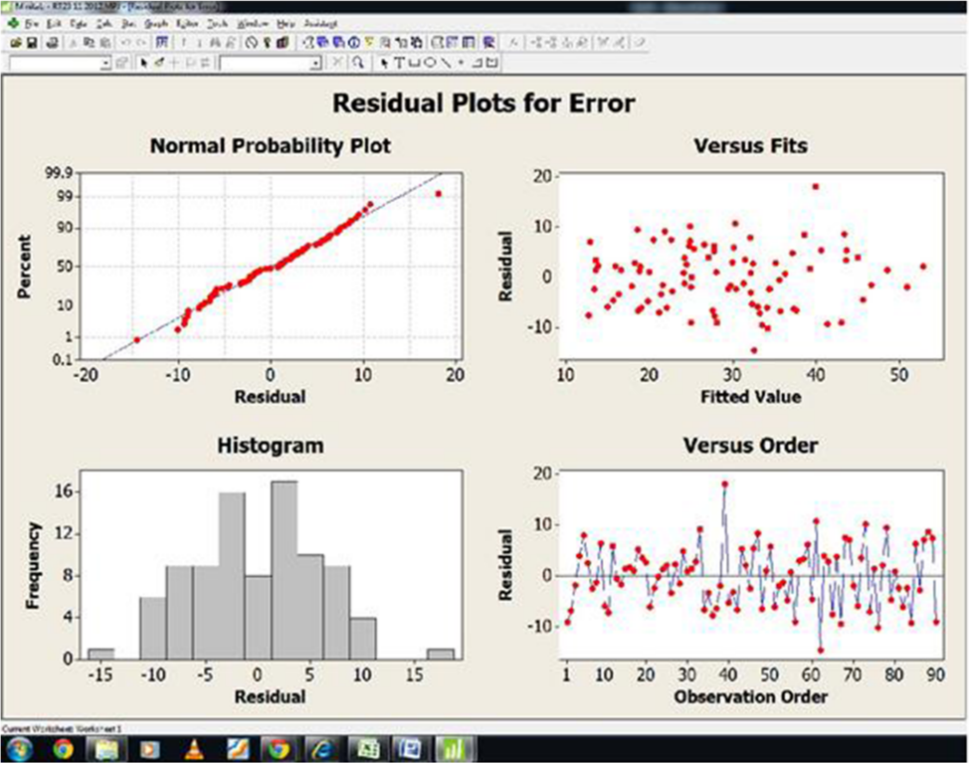
**Residual plots for error.**

Data has been collected from .NET software language database file. From this database file the response time and error rate was taken for analysis. The output total response time (Y_1_) of each volunteer has been noted in the Table 2 with respect to the treatment combination. Minitab 16 was used for statistical data analysis. 95% confident interval level maintained for the data analysis. Randomized block factorial design [16] was proposed for the NBT data analysis. Tables 4 and 5 shows all the independent and combined effect of temperature, and illumination for reaction time response (Y_1_) and Error (Y_2_) respectively. Figures 3 and 4 shows the residual plots for reaction time and error.

**Table 4:** **Test result for reaction time (Y**_**1**_ **)**

**Source**	**F**_**0**_	**F**_**0.05,v1,v2**_	**MS**	**F table value**	**Effect**	**% of contribution**
Temperature (A)	95.26	F_0.05,2,72_	101691.01	3.07	S	38.56
illumination (B)	49.19	F_0.05,2,72_	52515.81	3.07	S	19.91
AB	13.56	F_0.05,4,72_	14543.31	2.45	S	11.03
BLOCK	8.74	F_0.05,9,72_	9328.60	1.96	S	15.92

**Table 5:** **Test result for error (Y**_**2**_ **)**

**Source**	**F**_**0**_	**F**_**0.05,v1,v2**_	**MS**	**F table value**	**Effect**	**% of contribution**
Temperature (A)	32.79	F_0.05,2,72_	1428.08	3.07	S	25.05
illumination (B)	6.71	F_0.05,2,72_	292.04	3.07	S	5.12
AB	6.90	F_0.05,4,72_	300.41	2.45	S	10.78
BLOCK	9.24	F_0.05,9,72_	402.55	1.96	S	31.78

### Multi response performances

In this experiment two output responses such as Reaction time (Y_1_) and Error (Y_2_) have been investigated. The output reaction time and error for each volunteer can be recorded from the database folder. The multi response performance index was also be identified by using assignment of weights [17]. These two factors at each three level with two responses reaction time (Y_1_) and error (Y_2_) has been analyzed by assignment of weights method. In this method only 9 selected trials has been taken for analysis. These trials have been mentioned in the Table 6. The optimum level of each factors have also been identified for the best performance. The weights and Multi response performance index (MRPI) Values is shown in the Table 6. The level totals of MRPI values and optimum levels of each factor are shown in Table 7. The least error with short duration to complete the task was the objective to improve the performance. So “smaller the better” was followed for both the responses Y_1_ and Y_2_.

**Table 6:** **Weights and MRPI values for the experiment**

**Trial**	**Factors**		**Responses**		**weights of Y**_**1**_	**weights of Y**_**2**_	**MRPI**
	**A Levels**	**B Levels**	**Y**_**1**_	**Y**_**2**_			
1	1	1	5093	285	0.124724494	0.110294118	666.66
2	1	2	5579	415	0.136626341	0.160603715	828.89
3	1	3	4442	395	0.108781897	0.152863777	543.59
4	2	1	3968	262	0.097173924	0.101393189	412.15
5	2	2	4333	230	0.106112553	0.089009288	480.26
6	2	3	3394	209	0.08311701	0.080882353	299.00
7	3	1	5268	207	0.129010139	0.080108359	696.21
8	3	2	4431	259	0.108512514	0.100232198	506.78
9	3	3	4326	322	0.105941127	0.124613003	498.43

**Table 7:** **Level totals of MRPI and optimum level for each factor**

**Factors**	**Level**			**Optimum level**
	**1**	**2**	**3**	
Indoor room temperature	2039.13	*1191.41*	1701.41	*A*_*2*_
illumination	1775.01	1815.93	*1341.02*	*B*_*3*_

## Results

The results revealed that the independent and interaction effect of indoor room temperature and illumination have significant effect on the office workers’ performance. The effect of indoor room temperature has more influences than the effect of illumination. The effect of indoor temperature has 38.56% of contribution on the performance. The optimum level of indoor temperature at 21°C and illumination at 1000 lux has improved the work performance and health of office workers.

## Discussion

### Reaction time response (Y_1_)

The study result of reaction time indicates that the indoor room temperature has significant effect on the office worker’s performance. Similarly the effect of illumination also has significant effect on the performance of office workers. Referring to the Table 4, from the study result it may be noted that all the independent and combined effect of temperature, illumination have significant effect on the performance of office workers. Particularly the temperature has more significant effect than the effect of illumination. The effects of indoor room temperature have 38.56% of contribution on the office workers’ performance. Similarly the illumination has 19.91% influence of contribution on the performance of office workers. The combined effects of factors have 11.03% contribution on the performance of office workers.

### Error response (Y_2_)

From the Table 5, the indoor room temperature has 25.05% of contribution on office worker’s output error response. Similarly illumination has 5.12% of contribution on office worker’s performance. Interaction effect of indoor temperature and illumination has also significant effect (10.78%) on performance. Block effect of the error response has much more contribution (31.78%) on this experiment. This shows the volunteers were concentrating on time to complete the task, not concentrate on error. So the block effect was more.

### Multi response: reaction time and error response (Y_1_ and Y_2_)

The percentage of contribution of indoor room temperature much higher than the illumination on the both responses Y_1_ and Y_2_. Block effect has 31.78% of contribution in error response (Y2). Table 6 shows that the weights and MRPI Values for multi response Y_1_ and Y_2_. The Combined factors of optimum level A_2_B_3_ have least value of MRPI. Indoor room temperature of 2^nd^ level (A_2_ = 21°C), Illumination of 3^rd^ level (B_3_ = 1000 lux) given best performance of office workers. The least value of 299.00 (Table 6) was considered as optimum level of multi response.

## Conclusion

From the study result, the temperature and illumination are independently significant on the performance of the office workers have been understood. It may further be noted that the indoor room temperature has more significant effect than either independent or combined effect of illumination. From this, it may be concluded that the impact of indoor room temperature is more on the office worker’s performance than the effect of illumination. Block effect of the error response was more contribution on office worker’s performance. While considering the reaction time response, the block effect has very low contribution. But the block effects of both the responses Y_1_ and Y_2_ have significant effect.

From this, we can understand that the volunteers are more concentrate to do the NBT in short duration. So that reaction time response has less contribution of block effect obtained from this experiment. They were not cared about the errors. So the block effect was more in error response analysis. More over some of the volunteers were not wasting for thinking answer for unknown question in their test duration. They responded immediately to answer any one from the given choice. All the volunteers were completed the task before the time limit. But some of the volunteers had done more error in NBT. For this reason the block effect was more in error output response. The optimum level of this two factors (A_2_B_3_ ie., 21°C, 1000lux ) may improve the health and performance of office workers.
